# Quantifying the Performance of Micro-Compartmentalized Directed Evolution Protocols

**DOI:** 10.3390/life10020017

**Published:** 2020-02-13

**Authors:** Adèle Dramé-Maigné, Anton S. Zadorin, Iaroslava Golovkova, Yannick Rondelez

**Affiliations:** 1 Laboratoire Gulliver, ESPCI Paris, PSL Research University, 10 rue Vauquelin, 75005 Paris, France; 2 Laboratoire CBI, ESPCI Paris, PSL Research University, 10 rue Vauquelin, 75005 Paris, France

**Keywords:** directed evolution, enrichment factor, self-selection

## Abstract

High-throughput, in vitro approaches for the evolution of enzymes rely on a random micro-encapsulation to link phenotypes to genotypes, followed by screening or selection steps. In order to optimise these approaches, or compare one to another, one needs a measure of their performance at extracting the best variants of a library. Here, we introduce a new metric, the Selection Quality Index (SQI), which can be computed from a simple mock experiment, performed with a known initial fraction of active variants. In contrast to previous approaches, our index integrates the effect of random co-encapsulation, and comes with a straightforward experimental interpretation. We further show how this new metric can be used to extract general protocol efficiency trends or reveal hidden selection mechanisms such as a counterintuitive form of beneficial poisoning in the compartmentalized self-replication protocol.

## 1. Introduction

Molecular Directed Evolution (DE) is a technique used to obtain biomolecules with improved or novel functions, by iteratively generating pools of randomised variants and extracting the best performers from these pools. This technique, originally developed for the search of nucleic acid ligands [1,2], has been extended to proteins and used to explore other chemistries and functions [3,4]. Most importantly, it is now also used to select biopolymers with tailored catalytic properties, such as artificial enzymes or ribozymes [5,6].

The first high-throughput *enzyme* evolution experiments used living cells as a carrier and selection medium for the mutant phenotypes. In such approaches, the cell boundary ensures that the genotype-phenotype linkage is maintained, and that selection, acting on phenotypes, yields improved transferable genotypes. The selection is achieved by coupling the exogenous activity to the survival of the host cell in in vivo complementation methods. Alternatively, in so-called screening approaches, the cells are physically sorted according to an observable marker of the phenotype.

A number of limitations are associated with in vivo implementations. For example, some substrates may not efficiently penetrate the cell membrane, or it may be difficult to create a link between the desired catalytic activity and the cell survival. In many cases, toxicity effects associated with the exogenous activity also create a counter-selection pressure.

To bypass these limitations, a number of recent developments have introduced the idea of performing high-throughput directed enzyme evolution in vitro [7,8,9,10,11,12,13,14,15,16,17,18]. In these protocols, mutant genotype-phenotypes are randomly distributed in artificial micro-compartments, e.g., micro-droplets, with an internal volume ranging between femtoliters and nanoliters. As is the case for DE using living organism carriers, two strategies can be used to extract the best genotypes: *screening* and *selection*. In screening, the phenotypes produce a detectable modification of the physico-chemistry of the compartment, which is used to sort the compartments one-by-one into desirable and non-desirable bins [10,11,12,13,15,16,17,18]. In selection approaches, the artificial compartment autonomously enables a biochemical genetic amplification process, whose activation is conditional on the desired activity [7,8,9,14,18,19]. Importantly, high-throughput in vitro DE protocols generally use a *random* distribution of the mutant library in artificial micro-compartments (usually droplets, but also liposomes, micro-patterned arrays [20], etc.). In such approaches, there is no mechanism to ensure that one-and-only-one genotype will be present in each compartment. The experimenter only controls the *average* number of mutants per compartment (a value referred to as λ in the following discussion) but not their actual distribution in the compartment population. We are interested here in quantifying the performance of a given protocol, that is, its ability at enriching libraries in functional variants and eliminating non-functional ones. This performance depends on a number of experimental choices or constraints.

These include, in particular, the value of mean occupancy λ, but also the contamination by the parental genotype, the homogeneity of the compartment’s physicochemical parameters, the robustness of the genotype-phenotype linkage, experimental noise or errors, and so forth. In addition, high-throughput DE approaches are stochastic and multistep processes, making it challenging to properly assess their efficiency.

**Figure 1:**
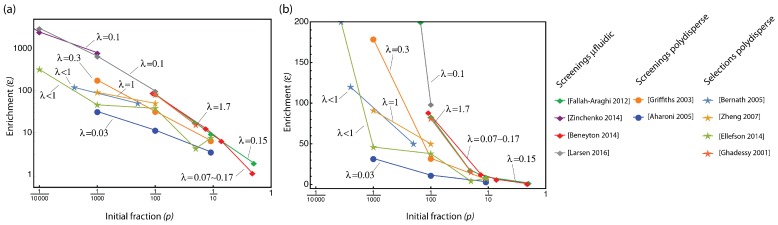
Enrichment factors vary over many orders of magnitude and have no direct interpretation. The enrichment factor from a selection of reports is plotted in log (**a**) or linear (**b**) scale against the initial fraction of active mutants used in the experiment. Lines connect data points from the same manuscript, and obtained with a single value of λ (the mean number of mutants per compartment, indicated next to the line). Disks are used for protocols using screening and polydisperse emulsions; diamonds, for protocols using screening and monodisperse (microfluidic) emulsions; stars, for protocols using selections in polydisperse emulsion. Some values were recomputed from the reported data.

The usual approach to assess efficiency is to perform a “mock selection” using a starting library comprising a known—and small—fraction of a functional variant in a pool of inactive variants. Previous works then directly compute a simple metric such as the “enrichment factor”, which is the ratio of the frequency of the active variant before and after selection. As we show below, such simple metrics however are, in fact, highly sensitive to experimental choices, lack generality, and may be in some cases misleading (Figure 1).

Here, we introduce an improved performance metric, the Selection Quality Index (SQI). Our approach is based on a mathematical analysis of compartmentalized selection processes, which includes, in particular, the effect of the experimentally controllable parameters: the mean occupancy λ, the initial fraction and the internal mechanism of the enrichment process. The SQI normalizes for these factors and therefore comes with an absolute interpretation. An SQI of 1 denotes an optimal protocol, whereas an SQI of 0 indicates a complete failure, i.e., the fact that cofounding effects have completely abolished enrichment (negative values indicate counter-selection). This index thus reflects the absolute potential of a given experimental approach to concentrate rare catalytic variants. We also show that it can be used to compare different experimental approaches or as a tool and guideline during protocol optimization.

## 2. Modeling High-Throughput Directed Evolution Protocols

### 2.1. Two Categories of Experimental Parameters

One cycle of a high-throughput in vitro DE protocol can be generically described by a sequence of three operations: First, the variant phenotypes are randomly distributed into micro-compartments. Second, a selection/screening process is applied *at the level of compartments,* by which genetic variants contained in highly active compartments are amplified (selection) or genetic variants from low activity compartments are removed (screening). While we discuss both cases here, the distinction between selections and screenings will be important in our analysis. Third, the compartments are pooled before a new cycle can take place.

The parameters necessary to describe such a protocol can be split in two groups, depending on whether they are controllable knobs of the experimental design or not. Parameters in group I (controllable) are in particular: the mean occupancy λ, the sharing status, and the replication/selection function. These factors, and the way they impact the selection efficiency, are presented below.

The mean occupancy λ, depends on the total concentration of variant in the starting mixture, with respect to the compartment size. At low mean occupancy, the chances of finding two co-compartmentalized phenotypes are very small, and clonality is high, but most of the compartments are empty. As λ increases, more and more compartments actually contain more than one phenotype, leading to the possibility of “hitch-hiking”: inactive mutants, which should not have survived the screening or selection process, are carried over by sharing a compartment with a better phenotype. In principle, one can infer the statistical distribution of occupancies from the compartmentalization process. When this process is random, the distribution follows a Poisson law. In this case, if, for example, λ is set to 1 (i.e., there are as many compartments as mutant genotypes), the proportions of compartments containing 0, 1, and more than one genotype will be roughly one third each. Lowering the value of λ decreases co-occupancy, but yields more wasted (unoccupied) compartments and thus a decrease in throughput.

The second design parameter, fitness sharing, describes what happens in compartments with co-encapsulated good and poor phenotypes. Two situations are possible. In the first one, typical of selection experiments, the genetic replication occurring in an active compartment is equally shared between all the co-encapsulated genotypes, whether or not they actually contributed to the phenotypic activity. In other words, the hitch-hiking poor genotype not only takes a free ride, but also negatively impacts the amplification of the good variant. The second case is more appropriate for screening protocols, which works by discarding the low activity genotypes, rather than replicating the good ones. In that case, an active variant contributes one genotype to the next generation, whether or not it was co-encapsulated with other variants. Therefore, the total number of active genotypes in the progeny is not affected by co-encapsulation—although, of course, their fraction is. These two situations are referred to as sharing and non-sharing, respectively, in the rest of the manuscript. Importantly, we assume here that phenotypes are additive. In particular, this means that a genotype always produces the same phenotype, whatever the other genotype it may be encapsulated with. Non-additive phenotype effects, which could arise, for example, from competition between co-compartmentalized genotypes for the phenotype-expression machinery, are explored in [21].

The third controllable factor is the replication/selection function, which we call *f*. This function describes how many genotypic copies are pushed to the next generation, according to the phenotypic activity observed in a compartment [22]. In screening, the value of the function is 1 if the phenotypic activity is above a given threshold, and 0 otherwise. In selection, it is set by the internal replication biochemistry and may take a variety of shapes. For example, a sigmoidal shape for *f* could encapsulate the fact that no replication happens when phenotypic activity is very low, that replication then improves with higher activity, and that the amount of material resources in the compartment sets a maximum for the number of genetic copies it can produce.

Group II consists of experimental idiosyncrasies that are not directly controllable. Together, they are responsible for the observed deviation from an optimal assay. These factors can be based on a variety of causes, some known or guessable, other unknown altogether. For example, in screening tests, the sorting machinery generally makes a number of errors, typically increasing when sorting is attempted at higher frequencies, or when the signal obtained from the droplets is weak [11]. Noise in expression can also play a role when compartments containing the same genotype may end up with various levels of phenotypic activity (typical when a bacterial expression system is used, i.e., in lysate assays [12]). Additionally, a leak between compartments, poor genotype-to-phenotype linkage (e.g., in mRNA display strategies), hidden selection biases (e.g., linked to toxicity effect during protein expression), non-homogeneity or polydispersity of the encapsulating compartments, carry-over of the parent genomes in selection protocols, and a myriad of other causes may affect the result. One does not typically have enough information to precisely describe or model these effects and is rather interested in quantifying their negative impact on the selection protocol, in order to try to optimize it empirically.

### 2.2. The Mathematical Model

We have recently shown that all effects belonging to group I can be modeled using a single equation, Equation (1). This equation describes the evolution of a population of variants subjected to random-compartmentalized selection rounds, characterized by a replication/selection function *f* acting on additive phenotypes, a sharing rule φ(n), and a distribution of phenotypes ρ [21].

(1) $$\rho^{\prime }=\frac{\sum_{n=0}^{\infty }\frac{\lambda^{n}}{n!}\varphi \left(n+1\right)\langle \delta_{x}*\rho^{*n},f\rangle }{\sum_{n=0}^{\infty }\frac{\lambda^{n}}{n!}\varphi \left(n+1\right)\langle \rho^{*n+1},f\rangle }\;\rho$$

where ∗ means convolution of distributions, $\delta_{x}$ is the delta-function centered at *x*, and 〈ρ,f〉 means application of the distribution ρ to the function *f*. φ(n) is the fraction of the value given by *f* allocated as copies to each variant in the droplet containing *n* variants. Note that this equation is valid under the assumption of random compartmentalization and additivity of phenotype. However, other cases can be treated by generalizations of this approach [21]. For example, the case of non-random compartmentalization is addressed at the end of this manuscript. Below, we will apply Equation (1) to evaluate the theoretical (expected) change in frequency during a test selection experiment, as a function of the parameters of group I.

### 2.3. Computing the Frequency Jump in Mock Libraries

**Figure 2:**
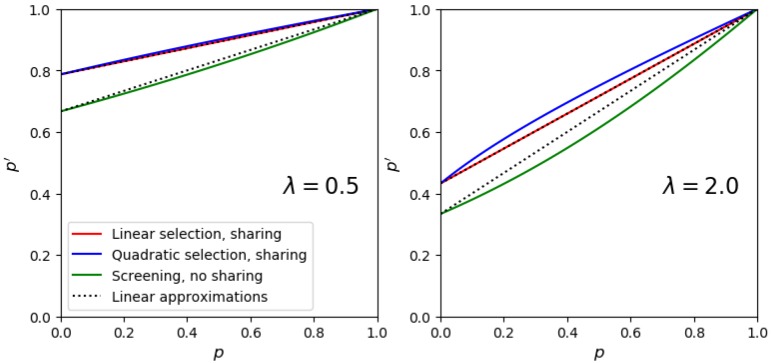
Expected new fraction $p^{\prime }$ as a function of initial fraction *p* for various replication/selection functions *f* and for two values of λ. The exact analytical results derived from (1) are shown by solid lines. The linear approximation is shown by a dotted line for the cases both with and without sharing. Δ, the frequency jump at very small initial fraction of active variant, corresponds graphically to the intercept of the curves with the vertical axis. As explained in the text, the theoretical value of Δ does not depend on the replication function *f*, allowing the derivation of general equations applicable to many protocols.

The usual way to test a DE protocol is to set up a mock experiment, where the initial library contains a known proportion *p* of active variants among many inactive ones. One then measures the proportion $p^{\prime }$ after a single selection round. In this case, the distribution of phnenotypes ρ has only two discrete values and Equation (1) can be used to provide the expected value of the new frequency $p^{\prime }$ as a function of the initial frequency *p* for different λ. We call this theoretical post-selection frequency $p_{\mathrm{theo}}^{\prime }\left(p\right)$ (Figure 2 and Supplementary Information). Importantly, this function is discontinuous at p=0, reflecting the effortless invasion of the population of dysfunctional mutants by a rare functional one.

In an ideal selection with no co-encapsulation, the jump observed at low *p*, corresponding to the selection of one active variant among an infinity of inactive ones should be equal to 1: all dysfunctional variants disappear and only the active ones remain. In a real experiment, this is unlikely to happen and one may instead observe an increase in frequency, but to a value smaller than one. Therefore, the value of the frequency jump, which we call Δ, provides a good absolute characterization of the selection efficiency at low *p* and depends only on λ. Indeed, one can show that Equation (1) yields an expression for Δ, which does not depend on the replication function *f*:

(2) $$\Delta =p_{\mathrm{theo}}^{\prime }\left(p\to 0\right)=\frac{\sum_{n=0}^{\infty }\frac{\lambda^{n}}{n!}\varphi \left(n+1\right)}{\sum_{n=0}^{\infty }\frac{\lambda^{n}}{n!}\left(n+1\right)\varphi \left(n+1\right)}.$$

Note that this proves that a correct quantification of selection is based on the difference between initial and final fractions, and not their ratio, as often used in enrichment factors. We see that all possible selection/screening scenarios collapse into only two cases: in the non-sharing situation, which is typical of screening protocols, we have φ(n)=1; in the sharing situation, which is more typical of selection assays, there is a cost associated with the presence of co-encapsulated variants and φ(n)=1/n. Equation (2) then simplifies to:

(3) $$\Delta_{\mathrm{screen}}=\frac{1}{1+\lambda }\;and\;\Delta_{\mathrm{sel}}=\frac{1-e^{-\lambda }}{\lambda }.$$

### 2.4. Definition of the Selection Quality Index (SQI)

Knowing λ, *p*, and the sharing behavior of a given experiment setup, one can thus obtain a good approximation of the expected maximal gene frequency Δ after one round. However, Δ is defined in the limit of vanishing *p*, which is not practical experimentally. We thus need the behavior of $p_{\mathrm{theo}}^{\prime }\left(p\right)$ close to 0. As this behavior does depend on *f*, we propose to correct it using a linear approximation of the following form.

(4) $$p_{\mathrm{theo}}^{\prime }\left(p\right)≃\Delta +\left(1-\Delta \right)p.$$

This expression is exact for linear replication functions and performs well for nonlinear ones (especially, for not very large λ, see Figure 2).

$p_{\mathrm{theo}}^{\prime }$ can now be used as a reference to assess the quality of the experimental protocol independently of the controllable parameters λ and *p*. In principle, one can thus use any convenient value of λ and *p* and simply measure the experimental frequency $p_{exp}^{\prime }$ after the selection cycle. The SQI of the protocol can then be expressed as the ratio of the experimental to theoretical frequency jumps, i.e:

(5) $$SQI=\left(p_{\exp}^{\prime }-p\right)/\left(p_{\mathrm{theo}}^{\prime }-p\right).$$

when replaced, we obtain:

(6) $$SQI_{\mathrm{screen}}=\frac{\left(1+\lambda \right)\left(p_{\exp}^{\prime }-p\right)}{1-p}\;SQI_{\mathrm{sel}}=\frac{\lambda e^{\lambda }\left(p_{\exp}^{\prime }-p\right)}{\left(e^{\lambda }-1\right)\left(1-p\right)}.$$

These formula provide an absolute measurement of protocol optimality, that corrects for the effects of *p*, λ, and the sharing behavior. It is independent of the replication/selection function *f*, and thus reflects only the experimental contingencies belonging to group II. Note that for an experiment using a mock library of known active fraction $p^{\prime }$, the measurement errors made in assessing the final fraction $p^{\prime }$ will reflect linearly in the SQI.

## 3. Results and Discussion

### 3.1. Looking at Experiments through SQI and Enrichment Factor

In an attempt to characterize the quality of a given high-throughput directed evolution protocol, most papers currently report an "enrichment factor" defined as $\varepsilon =\frac{p^{\prime }}{1-p^{\prime }}\cdot \frac{1-p}{p}$, where *p* and $p^{\prime }$ are the frequencies of the allele of interest before and after one selection cycle, respectively (in some cases the enrichment factor is defined using the simple ratio of frequencies, $\varepsilon =p^{\prime }/p$).

However, the usage of ε as a measure of quality comes with a number of issues, as illustrated by the compilation of reported values of ε from various protocols given in Figure 1 (the full tables are included in Supplementary Information Material & Methods, Tables S2–S4). First, ε depends strongly both on the initial fraction of functional mutants *p* and on the value of the mean occupancy λ. As can be seen in Figure 1, measurements of enrichment factors made with different values of *p* generally yield values of ε that vary over many orders of magnitude. Similarly, changes in λ yield different enrichment factors, with no clear interpretation. Second, the upper theoretical limit of ε is infinity, when *p* or λ become small. This reflects the fact that, in principle, a flawless selection or screening assay should get rid of the poor phenotype in a single round. It is therefore difficult to estimate what should be a correct, or acceptable, value of an experimental ε. In practice, we observe that smaller values of *p* tend to increase the observed value of ε, suggesting, against common knowledge, that all protocols perform better on libraries with less active variants. Additionally, measurements at very small λ are not useful in assessing a protocol meant to provide the highest possible throughput, as the throughput is actually proportional to λ. Overall, interpreting the enrichment factor is difficult and one clearly cannot use it to directly compare different protocols in terms of their ability to enrich libraries.

**Figure 3:**
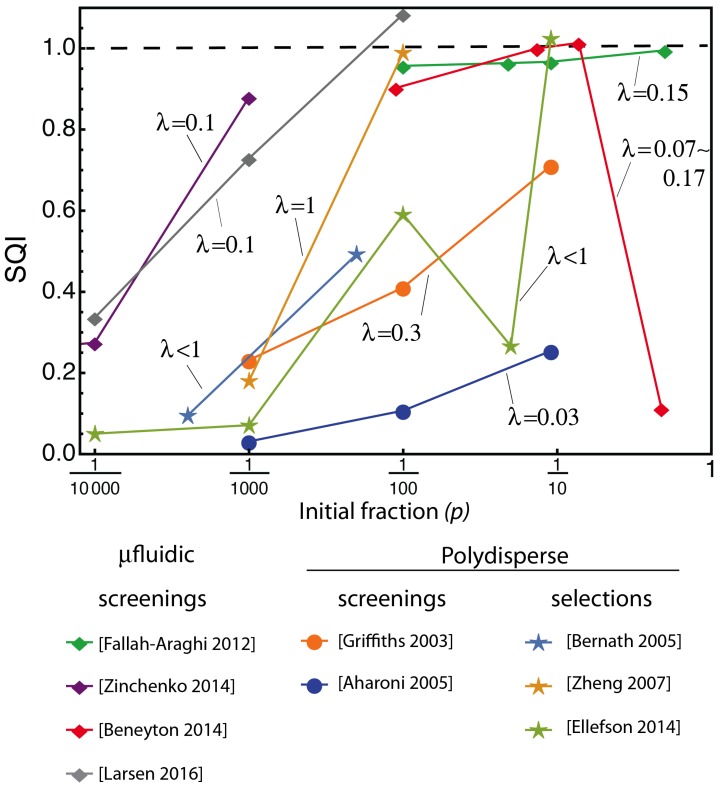
SQI calculated for the data in Figure 1. Lines connect data points originating from the same manuscript and done with a single value of λ (indicated next to the line). Disks are used for protocols using screening and bulk (non-monodisperse) emulsions; diamonds, for protocols using screening and microfluidic (monodisperse) emulsion; stars, for protocols using selections in bulk emulsion. The dotted line at SQI=1 represents the theoretical maximum performance, once the initial fraction and random partitioning have been taken into account. Note that the CSR (Compartmentalized Self-Replication) experiment [8] is off the scale here (see Figure 4).

In contrast, the SQI takes cofounding effects into account and provides a metric that is corrected for experimental parameters such as *p* and λ. In Figure 3, we use the reported data shown in Figure 1 to compute the SQI of a variety of experimental protocols and conditions. This immediately reveals some interesting trends.

First, some of the data series, for example the points from [Fallah-Araghi, 2012] [17] and [Beneyton, 2014] [16], taken at relatively high initial fractions (e.g., for p≥1/100), now give roughly the same SQI, independent of the initial fraction. This shows that the SQI metric is able to evaluate the intrinsic performance of an experimental protocol, independently of the controllable parameters. Additionally, for these relatively high initial fractions, we note a cluster of data points that achieve an SQI close to 1, indicating that the best achievable enrichment performance has been attained. Contrary to the enrichment factor, our metric takes into account the increased detrimental effect that is due to the co-encapsulation of variants at higher initial *p*, therefore revealing protocols that are experimentally optimal, although they were badly scored by the enrichment factor, which does not account for this effect. For suboptimal protocols, the value of the enrichment factor provide little clue about what to improve in the experimental design. In contrast, low SQI directly points at an issue with the processes and parameters from group II.

Second, for the lower *p* values, the selection efficiency is always decreasing with the decreasing initial fractions of active mutants. This stands in striking contrast with the trend observed in Figure 1, where enrichment factors seemed to indicate that all assays performed much better at lower initial fraction. This apparent trend is due to a problem in the definition of the enrichment factor, because lowering the initial *p* will necessarily lead to a higher value of ϵ at equivalent selection efficiencies. Our analysis corrects for that effect and reveals the fact that experimental contingencies dominate at low *p*, making experimental protocols less and less optimal as the initial fraction decreases. This can be due to a decreasing signal-to-background ratio, where the background noise can arise from a variety of phenomena, such as the contamination by parent DNA in selection protocols, sorting errors in screening protocols, genetic amplification biases, or the difficulty to recover minute amounts of genetic material in a general case.

Third, protocols can be separated according to their type: only screening using microfluidic compartmentalization appears to yield efficiencies close to 1. In contrast, protocols using screening of bulk emulsions typically have a lower performance. This indicates that the size-dispersity of the compartments is an important factor contributing to the efficiency of screening. Interestingly, some selection processes are able to obtain high indexes without resorting to monodisperse emulsion, which could be related to the superior robustness of selections versus screening, with respect to co-encapsulation (already noted in [22]). Indeed, reports concerning selections tend to use higher λ values.

### 3.2. The Compartmentalized Self-Replication (CSR) Protocol Has an Abnormal SQI

**Figure 4:**
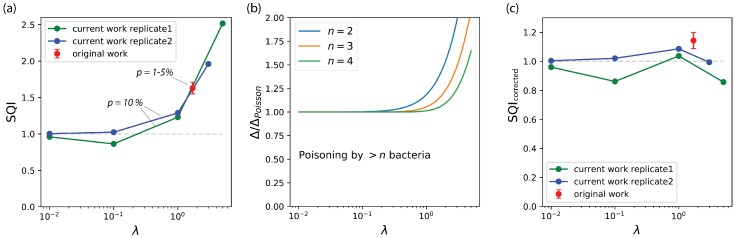
Compartmentalized self-replication (CSR) protocols are robust against co-encapsulation. (**a**): SQI calculated for CSR protocols at various λ. Red: original CSR report [8], in polydisperse emulsion. Blue and green: this work, two independent experimental replicates in monodisperse emulsions. The dotted line at SQI =1 represents the theoretical maximum efficiency. (**b**): Change in expected frequency jump assuming that droplets containing more than n bacteria are poisoned and do not participate in the reaction. (**c**): The SQI is recomputed with a cutoff set to n = 2, corresponding to the experimental observation that PCR is quickly poisoned by excess lysate.

The SQI appears able to stratify reported experimental platforms in terms of both the quality of the experimental design and their reaction to increasingly challenging libraries (that is, containing a decreasing fraction of active variants). One approach, however, appears to stand out in terms of its SQI, which is largely above 1 when measured for a high value of λ=1.7. This approach is an in vitro selection process, termed CSR, for compartmentalized self-replication [8]. It targets a bacterial library of variants of the Taq polymerase gene, which is randomly encapsulated in droplets with Taq-specific primers. After lysis at high temperature and PCR thermal cycling, mutant polymerases with higher activity replicate their own genes better than less active ones. When the droplets are broken, the retrieved genetic population is thus enriched in active Taq variants. According to its SQI, CSR apparently selects active mutants better than the theoretical limit associated with random co-encapsulation (Figure 4a, red point).

### 3.3. The Abnormal SQI of CSR Reveals a Hidden Beneficial Mechanism

The observation of SQI larger than 1 for CSR could indicate that this particular selection process has a built-in mechanism that limits the carryover of inactive variants. To investigate this point, and since the SQI in this case was evaluated from sparse reported information, we decided to re-implement the CSR experiment. Our initial attempts to reproduce the reported CSR protocol [8] failed, but we were able to reproduce the self-replication process with some modifications. First, we used Klentaq [23], a lysate-robust polymerase variant, instead of Taq. We found that this enzyme was expressed at sufficient levels in KRX cells (while the initial report had used TG1 cells). We produced an inactive variant by introducing the point mutation D332G. Finally, instead of the polydisperse emulsion in mineral oil initially reported, we generated monodisperse droplets in fluorinated oils, using a custom-made microfluidic flow-focusing step PDMS chip (see Supplementary Information Material & Methods). Provided these adjustments, we were able to observe the self-replication of the polymerase gene, both in bulk solution and in 24 μm droplets. This setup was used to perform mock selections and measure SQI for various values of λ. We measured the SQI of our modified version of CSR at different values of λ, as shown in Figure 4a. These measurements first showed that the CSR process is indeed able to perform optimally at low λ values, where the SQI is roughly 1. They also confirmed that higher λ results in SQI clearly above 1, and therefore that the CSR reaction is somehow immune to random co-encapsulation.

In the course of the experiments, we noted that the self-PCR reaction performed less efficiently when a higher concentration of bacteria was used in the master mix. This is most likely associated with the reported toxicity of bacterial lysates toward PCR [24]. We reasoned that this phenomenon could explain the observed behavior: if droplets containing more than one bacterium are unable to support PCR, whatever the mixture of active and inactive variants they contain, then the genes that are co-encapsulated in these droplets simply do not contribute to the new generation. Therefore, it is as if co-encapsulations were not occurring and the jump in gene frequency should be less affected (Figure 4b). To validate this hypothesis, we performed the self-PCR reaction, in test tubes, with mixtures of variants that reproduce the content of a droplet containing one active-variant bacterium, plus increasing amounts of inactive variants bacteria (see Supplementary Information Material & Methods). Indeed, we found that the self-PCR reaction yield decreased a lot as soon as a single inactive variant bacterium is co-encapsulated with an active one. The reaction does not happen at all with two or more encapsulated inactive bacteria. Figure 4c shows the recomputed SQI taking this phenomenon into account. The corrected SQI stays close to 1 for all experiments, irrespective of the value of λ. The CSR method thus performs optimally, at least around *p* = 1–10%, and has a built-in mechanism against hitch-hiking.

### 3.4. The Case of Non-Random Compartmentalization

**Figure 5:**
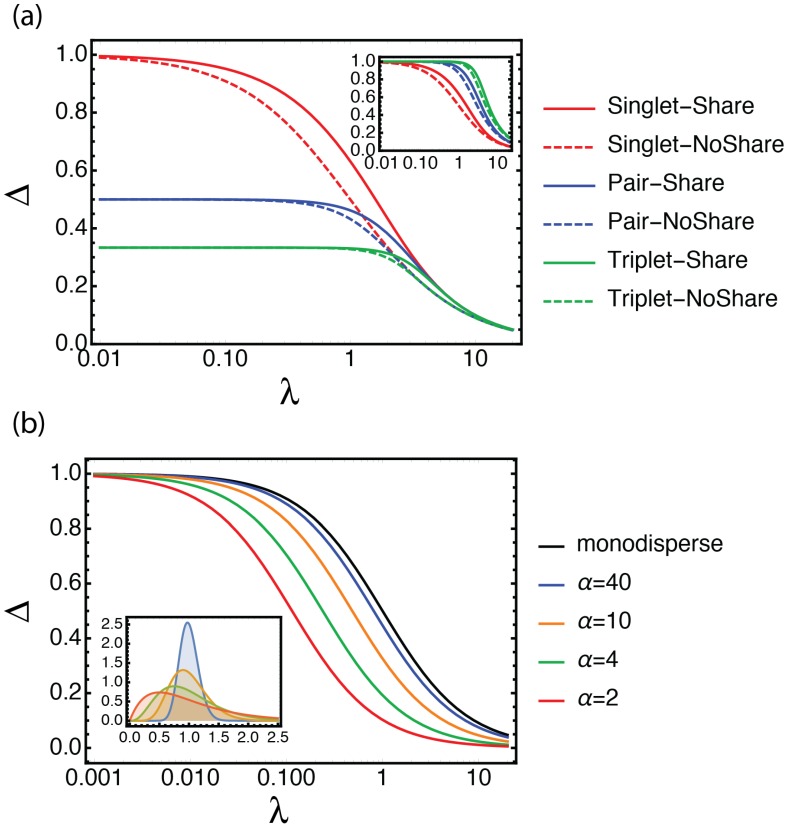
Non-Poissonian distributions in compartments. (**a**): The effect of aggregation of the mutants into pairs or triplets on the theoretical value of Δ (the achievable frequency jump in one round, valid for low *p*). Full lines correspond to sharing, while dotted lines indicate no sharing. Red; encapsulation of discrete individuals; blue, encapsulation in pairs; green, encapsulation in triplets. To show the effect of λ relative to the best possible frequency jump in a given aggregation condition (which happens at λ=0), we plot in inset, the rescaled curves $\Delta /\Delta_{\lambda =0}$. (**b**): The effect of polydispersity of the compartments in the case of screenings. Here, we assume that the volumes are Gamma-distributed with mean one and various shape parameters α. The corresponding distributions are shown in the inset.

The equations presented above are valid when individual mutants are *randomly* distributed in the available compartments. In this case, one expects a Poissonian distribution of the number of mutants per compartment, on which we have based our analysis. However, other distributions can be more relevant in some cases. For example, some microfluidic devices use physical effects to encapsulate objects in compartments with distribution sharper than the Poisson distribution [25,26,27,28]. Alternatively, mutant candidates may stick or aggregate to each other (for instance in pairs, triples, tetrads, etc.), which will also distort the distribution away from the Poisson law. Polyploidy represents a related case, although closer in context to population dynamics than to directed evolution protocols. Fortunately, our mathematical approach is general enough to handle many of these cases, and yield theoretical values of Δ in closed analytical form, when available. For example, Figure 5 shows the effect of aggregated partitioning in pairs or triplets (see Supplementary Information. When λ is small, we recover the intuitive result that the best possible outcome in terms of frequency jump is the inverse of the aggregation cluster size (i.e., in the case of *k*-clusters, the good variant can not invade more than 1/k of the population in one round). However, the negative effect of aggregation gets offset when λ increases, as all curves gather on the same asymptote, which depends only on the sharing behavior. Interestingly, we observe that the relative selection efficiently decreases faster for a singlet than for a doublet or triplets (Figure 5a, inset), indicating that clustered selections tend to be more resilient to increases in λ, compared to unclustered selections.

Many high throughput in vitro DE protocols use emulsions as a convenient way to provide compartments. Our bibliographic analysis has also highlighted the role of the mono/poly-dispersity of these emulsions on the selection process. At equal average compartment volume, polydisperse emulsions will have more compartments containing multiple mutants. This is expected to decrease their efficiency at selecting one particular phenotype from a mixture. Indeed, in the present case of selection from an active/inactive mixture of variants, we can show that a polydisperse emulsion always performs worse than a monodisperse one. This effect is shown for Gamma-distributed compartment volumes [29], in the case of screening, in Figure 5b.

## 4. Conclusions

We derived a robust metric that can be used to evaluate and compare in vitro high-throughput-directed evolution protocols. Like previous approaches, it simply requires a mock experiment, where a known mixture of functional and dysfunctional variants is subjected to one selection round, and the change in frequency is evaluated. Hence, it can directly be used by high-throughput-directed evolution practitioners without any change in their experimental practice and conveniently allows the re-evaluation of already existing or published data. However, contrary to its predecessors, our metric separates experimental design options into two categories, depending on whether they are easily tunable parameters or not. The predictable influences of the initial fraction *p*, the selection mechanism, as well as the average occupancy λ, are incorporated in the SQI, whose value reflects only the other, less controlled causes affecting the efficiency of the protocol.

When applied to reported data, the SQI provides an informed evaluation of protocol quality, with an SQI close to 1 indicating that the experimental procedure is close to optimal. Our approach allowed us to detect abnormal selection efficiency in the CSR, and we were able to link this behavior to a deviation from the intuitive model based on the additivity of the phenotypes. We have shown experimentally that this is most likely, due to the toxicity of bacterial lysate. Counter-intuitively, these toxic effects have a positive influence of the selection efficiency in CSR.

Because it corrects for controllable factors, the SQI is able to extract trends in between different experimental approaches, and suggests hypotheses about the causes behind the observed differences in efficiency. It can therefore help researchers to choose between protocols for their DE experiments. Overall, our re-analysis of existing protocols has shown that, whatever the experimental platform, SQI drops with decreasing initial fractions. This reflects the intuitive fact that is is more difficult to extract very rare variants. We suggest that this probably linked to a decrease in signal-to-background values, where the negative impact of experimental contingencies of type II become more prevalent.

For the experimentalist, the interpretation of an observed value of SQI depends on his expectation concerning the throughput of the protocol. For example, most recent micro-compartmentalized protocols target a throughput *T* between $10^{6}$ and $10^{9}$. It thus seems unlikely that a very rare active mutant can be purified in a single round. Many (mostly microfluidic) approaches already provide a good SQI at $p>10^{-3}$, implying immediate fixation in these cases. It is therefore the ability to bring a rare active variant—with an initial fraction that can be estimated as the inverse of the throughput of the protocol—to $p=10^{-3}$ that is going to be critical for real applications. We note that for small values of *p*, the SQI is simply the final fraction of active mutants, corrected for co-encapsulation and sharing/non sharing effects. This is because, at low *p*, according to Equation (5), the SQI can be written as

$$SQI=p^{\prime }/p_{\mathrm{theo}}^{\prime }\left(p\right)∼p^{\prime }/\Delta .$$

To estimate the number of necessary rounds, one should then attempt to measure the SQI at initial fractions close to 1/T. For example, if an SQI $>10^{-3}$ is obtained for *p* as low as 1/T, then one would typically need only two rounds to harness the full potential of the experimental protocol, that is, fix a functional variant initially present as a single copy in the library.

## Supplementary Materials

The following are available online at https://www.mdpi.com/2075-1729/10/2/17/s1. “Materials and Methods” and “Mathematical Analysis”.
