# Supplementary material for: Quantifying the Performance of Micro-Compartmentalized Directed Evolution Protocols
Source: Life (Basel). 2020 Feb 13;10(2):17. doi: 10.3390/life10020017 (PMC7175308; doi:10.3390/life10020017)
Supplement: Supplementary file 1 [file life-10-00017-s001.zip › SI/SI-material & Methods.pdf]

SI Material & Methods

Quantifying the performance of high-throughput directed evolution protocols

Adèle Dramé-Maigné*, Anton S. Zadorin*, Iaroslava Golovkova, and Yannick Rondelez

Mock selection of the KlenTaq polymerase

Compartmentalized selection with various λ

For mock selection experiments, an inactive version of the Klentaq polymerase was constructed via site-directed mutagenesis (Q5® Site-Directed Mutagenesis Kit from NEB) using the following primers:

Pr_NegMut_Fwd: 5′-GGTTGCACTGGGTTATAGCCAG

Pr_NegMut_Rw: 5′-AGCAGCCAACCTTCTTCGG

The aspartic acid 332 (GAT) was changed into a glycine (GGT) in the DYSQIELR motif (reference motif).

Bacteria expressing either a wild-type or the incativated version of the KlenTaq polymerase was grown and induced at 37°C and 250 rpm for 2 hours with 0.1% of L-rhamnose. After induction, the bacteria were pelleted in a centrifuge at 5000 g for 5min. They were washed (resuspended and centrifuged 5min at 5000 g) twice in a resuspension buffer (Tris-HCl ph 7.5 50mM, NaCl 100 mM). A solution was then prepared containing Thermopol DF buffer (NEB) (1x) with 1.5mM MgSO4, 400 µg/mL of BSA9000S (NEB), 200 µM of each dNTPs (NEB), 1ng/µL of Yeast RNA (Merck), 0.4% of Pluronic F-127 (Sigma-Aldrich) and the bacteria. We added 200nM of primers:

reverse: 5′-TTAGGTCTCACTAAGCAAAAAACCCCTC

forward: 5′-TTAGGTCTCATCTATAATACGACTCACTATAGGGAG

Bacteria concentration was determined using OD600 measurement. The ratio of active over inactive bacteria was 1:10. The premix was then injected in a microfluidic device under pressure control to generate droplets of ~24µm of diameters, with bacteria at indicated λ, in fluorinated oil (Novec 7500 (Sigma-Aldrich), 2% EA (Raindance)). The size of the droplets is controlled by a flow-focusing step junction at the nozzle. The collected droplets were then transferred in a PCR tube, and the PCR was initiated: 95°C 3min, (98°C 10s, 53°C 30s, 72°C 1 min 30) x 35, 72°C 2 min.

Selectivity assay

To assess the selectivity of the assay, rh qPCR reactions were run using IDT rhprimers and the RNaseH2. These primers allow to detect a single nucleotide mutation change. The rhPCR was set up using DreamTaq buffer (1x), 1.5mM of extra MgSO4 (NEB), 200µM of dNTPs (NEB), 1% Evagreen (Biotium), 0.5% of DreamTaq DNA polymerase (Thermofisher) and a specific amount of RNase H2 (IDT DNA technologies), 0.3 mU for the wild-type detecting reaction and 0.2 mU for the inactive klentaq detection in 10µL reacions, and 200nM of the two adapted rhPrimers:

forward wild-type: 5′-TTGGCTGCTGGTTGCACTGGrATTATC_3SpC3,

forward inactive: 5′-TTGGCTGCTGGTTGCACTGGrGTTATC_3SpC3

reverse: 5′-GCTTCACGCGGAACACCAAACrATCCAC_3SpC3

The 10 µL reactions were monitored in CFX96 Touch Real-Time PCR Machine (Bio-rad) following a 3 min denaturation at 95°C and 40 standard qPCR cycles (95°C 10 s, 60°C 40s). Standards were run

with plasmids of either active or inactive version of the gene starting from ~1 ng in 10 µL and diluted sequentially by 5 for 5 dilutions. To determine the proper amount of RNase H2 giving the best assay dynamic range, ranges of RNase H2 were realized for each primer couple. The results were compared to control reactions performed with the corresponding classical PCR primers.

Selection of KlenTaq active gene with various λ								
λ	Fraction (active/total)				Active genes (fraction) enrichment (n-fold)	SQI (Π)	Bulk final	
	initial	error	final	error			fraction	error
**1st selection experiment**								
**0,01**	**5,0%**		**96,0%**		**19,2**	**0.96**	31,7%	
**0,1**	**7,3%**		**84,0%**		**11,5**	**0.87**	20,7%	
**1**	**ND***		**80,0%**		**13,3**	**1.25**	57,9%	
**5**	**ND***		**55,0%**		**9,2**	**2.62**	24,1%	
**2nd selection experiment**								
**0,01**	**20,7%**	±6,9%	**99,9%**	±0,1%	**4,8**	**1.00**	24,63%	±10%
**0,1**	**19,3%**	±4%	**97,7%**	±0,7%	**5,1**	**1.02**	29,7%	±4,5%
**1**	**16,8%**	±3,5%	**83,3%**	±2,8%	**5,0**	**1.26**	36,6%	±11,4%
**3**	**24,0%**	±6,5%	**65,9%**	±9,5%	**2,7**	**1.74**	37,3%	±7%

* Values could not be determined experimentally. We chose to use 6% for calculations.

Table S1: Obtained initial and final active fraction by rhqPCR

Estimation of the effect of the co-encapsulation of active with inactive variants

A PCR was run in Thermopol DF buffer (NEB) (1x) with 1.5mM MgSO4, 400 µg/mL of BSA9000S (NEB), 200 µM of each dNTPs (NEB), 130 bact/nL (~1 bacteria per droplet) of bacteria expressing the active wild-type KlenTaq gene, and 200nM of reverse and forward primers:

reverse: 5′-TTAGGTCTCACTAAGCAAAAAACCCCTC

forward: 5′-TTAGGTCTCATCTATAATACGACTCACTATAGGGAG

Various amount of bacteria expressing the inactive KlenTaq mutant were added to simulate the encapsulation of the active wild-type with 1 and up to 8 inactive bacteria. PCR was run using the following protocol: 95°C 3min, (98°C 10s, 53°C 30s, 72°C 1 min 30) x 35, 72°C 2 min. Results presented on 1 show that the contamination of droplets containing one active bacteria with one inactive bacteria is greatly deacreasing the reaction yield. A slight amplification is visible with 2 inactive bacteria and then no gene product is observed.

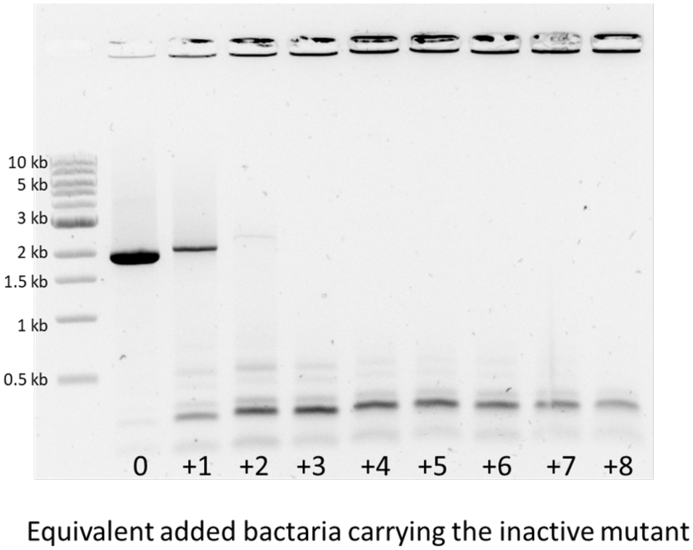

Figure 1: Assaying the effect of droplet contamination by inactive mutants. Self-Replication was run in test tubes with bacteria expressing the wild-type KlenTaq at a concentration equivalent to one bacteria per droplet (~130 bac/nL) and various amount of added bacteria expressing the mutant inactive KlenTaq (corresponding to the indicated equivalent contaminant inactive bacteria in a droplet).

Literature review

25 articles performing high-throughput selection or screening experiment were studied and the found characteristic parameters and results are recorded in table S2 S3 and S4 according to the emulsion generation technique. All values corresponding to the initial and final quantity of the active variant were converted in ratio of active over inactive clones when given in fraction originally.

#	Experiment (Target)	λ		Ratio active/inactive initial	final	Active genes enrichment (n-fold)	SQI (Π)	Achievement in xx rounds Throughput ()
**Screening experiments with emulsion generated by shaking or filter**								
**Several λ process : gene on beads, beads in IVTT, beads in selection droplets**								
1	Directed evolution of an extremely fast phosphotriesterase by in vitro compartmentalization, 2003 (Phosphotriesterase)	0.3		0.1	1.4	14	0.71	63 times higher k_{cat} in 6 rounds (6×10^8)
				0.01	0.48	47	0.41	
				0.001	0.21	217	0.23	
2	Ribozyme-Catalyzed Transcription of an Active Ribozyme, 2011 (Ribozyme polymerase)	?		0.1	~1000**	10^4	?	Polymerase ribozyme able to synthesize a wider spectrum of RNA sequence in 13 rounds ($\sim 5 \times 10^7$)
				0.001	~100**	10^5		
				10^{-5}	~1**	10^5		
**Encapsulation of cells (bacteria or yeasts)**								
3	High-Throughput Screening of Enzyme Libraries: Thiolactonases Evolved by Fluorescence-Activated Sorting of Single Cells in Emulsion Compartments, 2005 (Thiolactanase)	0.03	¤*gating*					100-fold improvement in activity of PON1 (paraoxonase) in 6 rounds ($\sim 5 \times 10^8$)
			0,0001	0.001	0.29	290	0.23	
				0.01	0.63	63	0.28	
			0,001	0.001	0.04	40	0.04	
				0.01	0.69	69	0.42	
			0,01	0.001	0	-	?	
				0.01	0	-	?	
4	Ultrahigh Throughput Screening System for Directed Glucose Oxidase Evolution in Yeast Cells, 2011 (Glucose oxidase)	0.01*				†*not defined*	?	1,8 increase in k_{cat} in 1 round ($\sim 10^7$)
				0.1		4,7		
				0.01		13		
				0.001		33		
**Encapsulation of genes by extrusion through filter**								
5	High-Throughput Screening of Enzyme Libraries: In Vitro Evolution of a β-Galactosidase by Fluorescence-Activated Sorting of Double Emulsions, 2005 (β-Galactosidase)	? (300)		0.33	1	3	?	Turn a protein of unknown function into β-Galactosidase in 2 rounds (4×10^7)
				0.008	0.82	103		
				0,008 enrich§	0.19	24		
				0,001	0.16	160		

* not explicitely given by the authors, estimated from available data in the paper

**estimated from gels given in the paper or sup mat by eyes

§ enrich is an alternative mode of screening, the other most use mode in this paper is called the purify mode

Table S2: Screening experiments with emulsion generated by shaking or filter

Screening experiments with emulsion generated by microfluidic							
#	Experiment	λ	Ratio active/inactive initial	Ratio active/inactive final	Active genes enrichment (n-fold)	SQI (Π)	Achievement in xx rounds Throughput ()
**Encapsulation of genes**							
6	A completely in vitro ultrahigh-throughput droplet-based microfluidic screening system for protein engineering and directed evolution, 2012 (β-Galactosidase)	0.15	1	13.84	14	0.99	In vitro screening method development (10^6)
			0.1	5.89	49	0.96	
			0.05	5.48	110	0.97	
			0.01	5.02	502	0.95	
7	Using droplet-based microfluidics to improve the catalytic properties of RNA under multiple-turnover conditions, 2014 (X-motif, ribozyme)	≤0.2	0.02		†*not defined* 28	?	k^{ss}_{cat} ~28-fold higher in 2 rounds (10^5-10^6)
**Encapsulation of cells (bacteria or yeasts)**							
8	Fluorescence-activated droplet sorting (FADS): efficient microfluidic cell sorting based on enzymatic activity, 2009 (β-Galactosidase)	0.021	0.1	213	2130	1.02	Screening method development ($2x10^3$ per second)
		0.016	0.5	537	537	1.01	
		0.16	0.01	1.35	135	0.66	
		0.2	0.1	7	70	1.03	
		0.15	0.5	16.1	16	0.78	
		1.6	0.01	0.294	30	0.57	
		0.91	0.1	0.553	6	0.55	
9	One in a Million: Flow Cytometric Sorting of Single Cell-Lysate Assays in Monodisperse Picolitre Double Emulsion Droplets for Directed Evolution, 2014 (Arylsulfatase)	0.1	0.001	4	4000	0.88	New screening system (>10^8 per day)
		0.1	0.0001	0.33	3333	0.27	
		1	0.000001	0,11	111 111	0.20	
10	Evolution of enzyme catalysts caged in biomimetic gel-shell beads, 2014 (Phosphotriesterase)	?	0.0001		†*not defined* > 100000 (in 2 rounds)	?	20-fold increase in speed in less than one hour in 1 round (>10^7 per hour)
11	CotA laccase: high-throughput manipulation and analysis of recombinant enzyme libraries expressed in E. coli using droplet-based microfluidics, 2014 (Oxidoreductase)	0.17	0.16	7.68	48	1.01	Screening method development (10^6 cells in 1,5 hours (with λ =0,5))
		0.135	0.009	3.93	437	0.90	
		0.071	0.08	15.28	191	1	
		0.1	0.9	1.12	1.24	0.12	

Table S3 - PART 1: Screening experiments with emulsion generated by microdfluidics

Screening experiments with emulsion generated by microfluidic							
#	Experiment	λ	Ratio active/inactive initial	final	Active genes enrichment (n-fold)	SQI (Π)	Achievement in xx rounds Throughput ()
**Encapsulation of cells (bacteria or yeasts)**							
12	High-throughput screening for industrial enzyme production hosts by droplet microfluidics, 2014 (Amylase)	?	0.25	3.5	14	?	2-fold increase in α-amylase production in 1 round (10^5)
13	A high-throughput cellulase screening system based on droplet microfluidics, 2014 (Cellulase)	?	0.001	0.43	430	?	Method development
			0.014	1.75	125		
			0.078	7.62	97.7		
			1	21.7	21.7		
14	Dissecting enzyme function with microfluidic-based deep mutational scanning, 2015 (β-Glucosidase)	0.1	0.54	49	90.7	1.07	Deep mutational scanning (10^7)
15	Ultrahigh-throughput discovery of promiscuous enzymes by picodroplet functional metagenomics, 2015 (Detection Phosphotriesterase)	0.8	4.6×10^{-9}	2.3×10^{-5}	$\sim5\times10^{3}$	4.1×10^{-4}	Identification of starting point sequences for new functions in 3 rounds (10^7-10^8)
16	High-throughput screening of filamentous fungi using nanoliterrange droplet-based microfluidics, 2016 (α-amylase)	0.24	0.21	40.7	196	1.2	2,3 fold activity increase in 1 round (5×10^4)
17	Ultrahigh-throughput–directed enzyme evolution by absorbance-activated droplet sorting (AADS), 2016 (Oxydoreductase)	1	0.0002	1.27	6350	1.12	activity >4.5-fold, kcat >2.7-fold, soluble expression 60% higher, Tm 12 °C higher in 2 rounds (10^6)
18	A general strategy for expanding polymerase function by droplet microfluidics, 2016 (Polymerase)	0.1	0.01	70.4**	~4 500	1.08	TNA polymerase in 1 round (3.6×10^7)
			0.001	2**		0.8	
			0.0001	0.44**		0.4	

**estimated from gels given in the paper or sup mat by gel analysis with ImageJ

† not defined : the formula of the enrichment is not given

Table S3 - PART 2: Screening experiments with emulsion generated by microdfluidics

Selection experiments with emulsion generated by shaking							
#	Experiment	λ	Ratio active/inactive initial	Ratio active/inactive final	Active genes enrichment (n-fold)	SQI (Π)	Achievement in xx rounds Throughput ()
**Encapsulation of genes**							
19	Man-made cell-like compartments for molecular evolution, 1998 (Methyltrasnferase)	1	0.001	1	1000	0.79	Method development (10^{10})
20	Directed Evolution of Protein Inhibitors of DNAnucleases by in Vitro Compartmentalization (IVC) and Nano-droplet Delivery, 2005 (Inhibition of self-DNA destruction)	≤1	0.005	0.33	66	?	claim >100 fold ≤ 500 fold, changing protein target in 8 rounds (~10^{10})
			0.001	?			
			0.0004	0.05	125		
21	Selection of restriction endonucleases using artificial cells, 2007 (Restriction endonuclease)	1	0.001	>0.1	≥100		20-fold improvement of activity in 3 rounds (10^{10})
			0.01	1	100	0.78	
22	An in vitro Autogene, 2012 (T7 RNA polymerase mRNA)	0.01-1	0.11	4	36	?	Too many mutations arise in 4 rounds (10^{8}-10^{10})
**Encapsulation of bacteria**							
23	Directed evolution of polymerase function by compartmentalized self-replication, 2001 (Taq Polymerase)	1.7*	0.01-0.05	4.3-3.3	109	1.63	11-fold higher thermostability, 130-fold resistance to inhibitors in 3 rounds ($2x10^{8}$)
24	Directed evolution of genetic parts and circuits by compartmentalized partnered replication, 2014 (tRNA synthetase)	≤1?	0.1	2.1**	21	?	T7 RNA polymerase orthogonal promoter (40-60% WT promotor)+ unnatural amino acids incorporation by tryptophanyl tRNA-synthetase: suppressor tRNA (1500-fold) in 10 to 16 rounds (>10^{6})
			0.01	0.6**	60		
			0.001	0.05**	50		
			0.0001	0.033**	330		
			0.00001	-			
			0.05	0.27	5.4	?	
**Encapsulation of complexes**							
25	Compartmentalization of destabilized enzyme–mRNA–ribosome complexes generated by ribosome display: a novel tool for the directedevolution of enzymes, 2013 (Reverse Transcriptase)	0.27	0.02	1	50	0.56	3-fold activity increase, thermoresistance in 5 rounds (10^{10})

* not explicitely given by the authors, estimated from available data in the paper
**estimated from gels given in the paper or sup mat by gel analysis with ImageJ

Table S4: Selection experiments with emulsion generated by shaking
