# Supplementary material for: Quantifying the Performance of Micro-Compartmentalized Directed Evolution Protocols
Source: Life (Basel). 2020 Feb 13;10(2):17. doi: 10.3390/life10020017 (PMC7175308; doi:10.3390/life10020017)
Supplement: Supplementary file 1 [file life-10-00017-s001.zip › SI/SI_Math.pdf]

Supplemental text
Mathematical analysis
Quantifying the performance of micro-compartmentalized directed evolution protocols

A. Drame Maigne, A. S. Zadorin, I. Golovkova, Y. Rondelez

February 11, 2020

Contents

1 Enrichment factor is not good for characterization of the selection efficiency

In applications, a popular measure of the selection strength is the so called enrichment factor $\varepsilon \stackrel{\text{def}}{=} \frac{p'}{1-p'} \cdot \frac{1-p}{p}$, where p' is the frequency of the allele of interest after one selection cycle. This value is often used to characterized the efficiency of the enrichment of a functional mutant in a population of dysfunctional wild ones—a typical situation in screening. In this section, we will examine expressions for ε in case of linear and cut-off selection function with or without sharing. These expressions are derived from the update equations for these cases taken from [1].

With the assumption of a population of a functional and a dysfonctional mutants, the linear replication function results in the following expression for ε

$$\varepsilon = 1 + \frac{g(\lambda)}{1 - g(\lambda)} \frac{1}{p},$$

where

$$g(\lambda) \stackrel{\text{def}}{=} \frac{1 - e^{-\lambda}}{\lambda}.$$

As expected, $\varepsilon \to \infty$, when $\lambda \to 0$, and $\varepsilon \to 1$, when $\lambda \to \infty$. In agreement with the linearizations, at $p \to 0$ we have $\varepsilon \to \infty$ and at $p \to 1$ we have $\varepsilon \to 1/(1 - g(\lambda)) = \beta^{-1}$, where β is the corresponding multiplier of the linearization of the update equation at $p = 1$.

The linear selection without sharing results in an even simpler expression

$$\varepsilon = 1 + \frac{1}{\lambda p}.$$

The cut-off selection without sharing corresponds to

$$\varepsilon = \frac{1}{1 - e^{-\lambda p}}.$$

Again, $\varepsilon \to \infty$, when $\lambda \to 0$ and $p \to 0$, $\varepsilon \to 1$, when $\lambda \to \infty$, and when $p \to 1$ we have $\varepsilon \to 1/(1 - e^{-\lambda})$, which is β^{-1} for this case. This expression was originally derived in [2].

Likewise, the cut-off selection with sharing results in

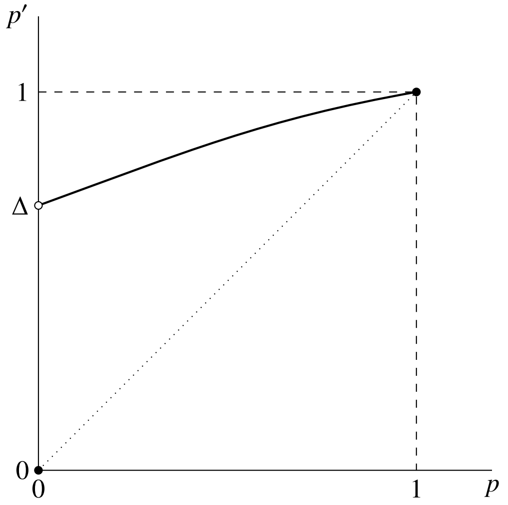

Figure 1: A sketch of a typical function $p'(p)$ (the thick line) for a fixed value of λ, $0 < \lambda < \infty$, for a mixed population of a functional mutant with frequency p and of a dysfunctional one with frequency $1 - p$.

$$\varepsilon = \frac{(1-p)\left(e^{\lambda}-1\right)}{(1-p)e^{\lambda}+p-e^{\lambda(1-p)}}.$$

As before, $\varepsilon \to \infty$, when $\lambda \to 0$ and $p \to 0$, $\varepsilon \to 1$, when $\lambda \to \infty$, and $\varepsilon \to \beta^{-1}$, when $p \to 1$, where $\beta = 1 - 1/g(-\lambda)$.

In fact, in general $\varepsilon \to \alpha$, when $p \to 0$, and $\varepsilon \to \beta^{-1}$, when $p \to 1$, where α is the multiplier of the linearization of the update equation in the vicinity of $p = 0$. As ε depends on p, it is not a good measure of the efficiency of the selection process by itself. But even a pair (p, ε) does not well characterize the selection, as the dependence of ε on p may be very different, as it is seen from the examples above. A complete characterization would be given by the entire curve $\varepsilon(p)$. However, gathering this information is very costly experiment-wise. An alternative could be the measurement of the asymptotic behaviour at $p = 0$ and at $p = 1$ and some interpolation in-between.

In reality, when a rare functional mutant is selected from a population of dysfunctional ones, only the asymptotic at $p = 0$ is relevant. Furthermore, as we have seen, the multiplier α of the linearization at this point is not informative, as the linearization itself is not defined ($\alpha = \infty$). This happens because the curve $p'(p)$ becomes discontinuous at $p = 0$. Although $p'(0) = 0$, the limit $\Delta \stackrel{\text{def}}{=} \lim_{p\to 0} p'(p)$ is positive, which reflects the effortless invasion of the population of dysfunctional mutants by a functional one. Therefore, this jump Δ is itself a good characteristics of the selection efficiency in such a case (see Figure 1).

The corresponding asymptotic of $p'(p)$ at $p \to 0$ up to the linear term is given below for all the cases we discussed before. The linear selection function with sharing:

$$p' = g(\lambda) + \left(1 - g(\lambda)\right)p. \tag{1}$$

The linear selection without sharing:

$$p' = \frac{1}{1+\lambda} + \frac{\lambda}{1+\lambda}\,p. \tag{2}$$

The cut-off selection without sharing (screening):

$$p' = \frac{1}{1+\lambda} + \frac{\lambda(2+\lambda)}{2(1+\lambda)^2}p + o(p). \tag{3}$$

The cut-off selection with sharing:

$$p' = g(\lambda) + \frac{1 - e^{-\lambda}}{2} p + o(p). \tag{4}$$

2 Initial jump Δ and efficiency comparison

Equations (1–4) show an interesting tendency. The value of Δ does not depend on the replication function. It only depends on the offspring sharing rule. It appears that this is a general property.

For a general replication function f, a general sharing rule $\varphi(n)$ (the fraction of the value given by f allocated to each individual in the droplet of size n), and a general phenotypic distribution ρ, the update equation is given by (see [3])

$$\rho' = \frac{\sum_n P_n n \varphi(n) \langle \delta_x * \rho^{*n-1}, f \rangle}{\sum_n P_n n \varphi(n) \langle \rho^{*n}, f \rangle} \rho.$$

Here probability distributions are treated as generalized functions (Schwartz' distributions, linear continuous functionals on continuous functions with finite support) and $\langle \rho, \varphi \rangle$ means the action of distribution ρ on function φ. The asterix means convolution and ρ^{*n} means the nth convolutional power. δ_x means δ-function concentrated at x. P_n is the probability to find n individuals in a compartment.

In the case of a mix of a lethal phenotype and a functional one with value x, we have $\rho = (1 - p)\delta_0 + p\delta_x$, $\rho' = (1 - p')\delta_0 + p'\delta_x$, $f(0) = 0$, and we are interested in looking at the term with δ_x at the next step of selection to determine p'. One can show that in this case, for f that does not grow too steeply, we have the following limit

$$\frac{\sum_n P_n n \varphi(n) \langle \delta_x * \rho^{*n-1}, f \rangle}{\sum_n P_n n \varphi(n) \langle \rho^{*n}, f \rangle} p\delta_x \to \frac{\sum_n P_n n \varphi(n)}{\sum_n P_n n^2 \varphi(n)} \delta_x, \quad p \to 0.$$

And therefore, we have, for a general partition rule P_n,

$$\boxed{\Delta = \frac{\sum_n P_n n \varphi(n)}{\sum_n P_n n^2 \varphi(n)}.} \tag{5}$$

For the Poisson law $P_n = e^{-\lambda}\lambda^n/n!$ we have

$$\boxed{\Delta = \frac{\sum_{n=0}^{\infty} \frac{\lambda^n}{n!} \varphi(n+1)}{\sum_{n=0}^{\infty} \frac{\lambda^n}{n!} (n+1)\varphi(n+1)}.} \tag{6}$$

This stays true even for a nonadditive mixing of activities (provided sufficiently limited growth of the replication function with the number of individuals in a droplet and with their activities). Here the update equation takes the form (see [3])

$$\rho' = \frac{\sum_n P_n n \langle \delta_x \otimes \rho^{\otimes n-1}, f_n \rangle}{\sum_n P_n n \langle \rho^{\otimes n}, f_n \rangle} \rho. \tag{7}$$

Here $f_n \colon \mathbb{R}^n \to \mathbb{R}$ are the functions that encode return the per-individual fitness to n phenotypic activities of coencapsulated individuals and $\otimes$ means the tensor product of distributions.

If we now assume $\rho = (1-p)\delta_0 + p\delta_x$, $f_n(0,\dots,0) = 0$, $f_n(x,0,\dots,0) = \varphi(n)$, then we recover (5) in the limit $p \to 0$.

For the selection with sharing we have $\varphi(n) = 1/n$, and thus (6) gives $\Delta = g(\lambda)$, regardless of f. Without sharing we have $\varphi(n) = 1$, and thus $\Delta = 1/(1+\lambda)$. In the case of sharing both the activity and the offspring, we have $\varphi(n) = 1/n^2$, and thus $\Delta = (\mathrm{Ei}(\lambda) - \ln\lambda - \gamma)/(e^\lambda - 1)$, where Ei is the exponential integral and γ is Euler's constant.

We can conclude that, if the sharing behaviour is known in the experiment, one can obtain the theoretically expected ideal macroscopic gene frequency jump Δ after one round of selection of a very rare functional mutant in a population of dysfunctional ones. This Δ can be used as a benchmark for the comparison of the experimental protocol efficiency. The advantage of this value is its independence of the replication function f.

3 Examples with P_n different from the Poisson law

We will illustrate how to apply (5) to some cases of non-Poissonian partitioning P_n. In particular, we will show how the well known case of a polyploid population (with no recombination) is recovered from the general formula, we will also discuss what changes if only pairs, triples, etc. are randomly encapsulated. The latter situation corresponds either to 'sticking' of haploid individuals (for instance in pairs, triples, tetrads, etc.) or to random encapsulation of polyploid individuals, when the total droplet phenotype depends only on the total chromosomal composition regardless the origin of each chromosome. Finally, we will consider the inhibition of replication by multiple encapsulation.

3.1 Classical selection of polyploid organisms

Consider $P_n = \delta_{kn}$, where δ_{kn} is the Kronecker delta. This means that each droplet contains exactly k individuals. In this case, the droplets effectively behave as k-ploid organisms, where individuals play role of (single locus) chromosomes inside droplets and that of gametes during the redistribution.

It is intuitively clear what to expect from the selection of a rare functional mutation in such situation. Indeed, any droplet that contains a functional gene also contains $k-1$ dysfunctional ones in the limit $p \to 0$. Therefore, after selection we will have $p' = 1/k$, and thus, $\Delta = 1/k$. As expected, when $P_n = \delta_{kn}$ is substitudet to (5), we get the same result regardless of φ:

$$\Delta = \frac{k\varphi(k)}{k^2\varphi(k)} = \frac{1}{k}. \tag{8}$$

3.2 Random compartmentalization of k-tuplets

The case of a random compartmentalization of k-tuplets of individuals corresponds to

$$P_n = \begin{cases} \mathcal{N}\dfrac{\lambda^n}{n!}, & n \equiv 0 \mod k, \\ 0, & n \not\equiv 0 \mod k, \end{cases}$$

where $\mathcal{N}$ is the normalization constant. Let us also denote $\tilde{P}_n \stackrel{\text{def}}{=} P_n/\mathcal{N}$. It is clear, that Δ can also be computed as

$$\Delta = \frac{\sum_n \tilde{P}_n n\varphi(n)}{\sum_n \tilde{P}_n n^2\varphi(n)}. \tag{9}$$

To find the expression for Δ with both $\varphi(n) = 1/n$ (sharing) and $\varphi(n) = 1$ (no sharing), it is enough to find

$$\Sigma \stackrel{\text{def}}{=} \sum_n \tilde{P}_n, \quad \langle n\rangle \stackrel{\text{def}}{=} \sum_n \tilde{P}_n n, \quad \text{and} \quad \langle n^2\rangle \stackrel{\text{def}}{=} \sum_n \tilde{P}_n n^2.$$

(As a matter of fact, $\mathcal{N} = \Sigma$.) Then Δ can be written in the following way:

$$\Delta = \frac{\Sigma - 1}{\langle n \rangle} \text{ with sharing;} \quad \Delta = \frac{\langle n \rangle}{\langle n^2 \rangle} \text{ without sharing.}$$

The following expressions, which can be proved by termwise differentiation of Σ in λ, are also useful:

$$\langle n \rangle = \lambda \frac{d}{d\lambda} \Sigma, \quad \langle n^2 \rangle = \left(\lambda^2 \frac{d^2}{d\lambda^2} + \lambda \frac{d}{d\lambda} \right) \Sigma. \tag{10}$$

3.2.1 Compartmentalization in pairs ($k = 2$)

In this case we have

$$\Sigma = \sum_{n=0}^{\infty} \frac{\lambda^{2n}}{(2n)!} = \operatorname{ch} \lambda, \tag{11}$$

where $\operatorname{ch} x \overset{\text{def}}{=} (e^x + e^{-x})/2$ is the hyperbolic cosine. Using (10), we find

$$\langle n \rangle = \lambda \operatorname{sh} \lambda, \quad \langle n^2 \rangle = \lambda^2 \operatorname{ch} \lambda + \lambda \operatorname{sh} \lambda,$$

where $\operatorname{sh} x \overset{\text{def}}{=} (e^x - e^{-x})/2$ is the hyperbolic sine. Therefore, we have

$$\Delta = \frac{\operatorname{ch} \lambda - 1}{\lambda \operatorname{sh} \lambda} \text{ with sharing;} \quad \Delta = \frac{\operatorname{th} \lambda}{\lambda + \operatorname{th} \lambda} \text{without sharing,} \tag{12}$$

where $\operatorname{th} x \overset{\text{def}}{=} \operatorname{sh} x / \operatorname{ch} x$ is the hyperbolic thangent.

3.2.2 Compartmentalization in triplets, quadruplets, etc. ($k > 2$)

For a given k consider complex kth roots of unity, that is complex numbers z such that $z^k = 1$. There are exactly k different roots and they all have the form $z = e^{2\pi i m/k}$, where $m \in \mathbb{Z}$ and i is the imaginary unit (of course, only $m \in \{0, 1, \ldots, k-1\}$ are essential). They posses a simple but useful property valid for any $l \in \mathbb{Z}$:

$$\sum_{m=0}^{k-1} \left(e^{2\pi i m/k} \right)^l = 1 + e^{2\pi i l/k} + e^{2\pi i \cdot 2l/k} + \ldots + e^{2\pi i (k-1) l/k} = \begin{cases} k, & l \equiv 0 \mod k, \\ 0, & l \not\equiv 0 \mod k. \end{cases}$$

This property allows to express $\tilde{P}_n$ in the following form

$$\tilde{P}_n = \frac{1}{k} \sum_{m=0}^{k-1} \frac{\left(\lambda e^{2\pi i m/k} \right)^n}{n!}.$$

Now Σ splits into k individual sums each of which results in a simple exponential function

$$\Sigma = \frac{1}{k} \sum_{n=0}^{\infty} \sum_{m=0}^{k-1} \frac{\left(\lambda e^{2\pi i m/k} \right)^n}{n!} = \frac{1}{k} \sum_{m=0}^{k-1} e^{\lambda e^{2\pi i m/k}}$$

In fact, this was already secretly used in (11), where we have $\{e^{2\pi i m/2}\} = \{1, -1\}$, and thus, $\Sigma = (e^{\lambda} + e^{-\lambda})/2$. Using (10), we find

$$\langle n \rangle = \frac{\lambda}{k} \sum_{m=0}^{k-1} e^{2\pi i m/k} e^{\lambda e^{2\pi i m/k}}, \quad \langle n^2 \rangle = \frac{\lambda^2}{k} \sum_{m=0}^{k-1} e^{4\pi i m/k} e^{\lambda e^{2\pi i m/k}} + \frac{\lambda}{k} \sum_{m=0}^{k-1} e^{2\pi i m/k} e^{\lambda e^{2\pi i m/k}}.$$

Finally, the the general expression for Δ in case of an arbitrary k is

$$\Delta = \frac{\displaystyle\sum_{m=0}^{k-1} e^{\lambda e^{2\pi i m/k}} - k}{\displaystyle\lambda \sum_{m=0}^{k-1} e^{2\pi i m/k} e^{\lambda e^{2\pi i m/k}}} \text{ with sharing;} \quad \Delta = \frac{\displaystyle\sum_{m=0}^{k-1} e^{2\pi i m/k} e^{\lambda e^{2\pi i m/k}}}{\displaystyle\sum_{m=0}^{k-1} e^{2\pi i m/k} e^{\lambda e^{2\pi i m/k}} \left(1 + \lambda e^{2\pi i m/k}\right)} \text{ without sharing.} \qquad (13)$$

It is not difficult to show that $\Delta \to k^{-1}$, as $\lambda \to 0$ (reduction to the k-ploid case (8)), and $\Delta \sim \lambda^{-1}$, as $\lambda \to \infty$. It is also easy to check that the cases $k = 1$ (the Poisson partitioning) and $k = 2$ (the Poisson partitioning of pairs) are correctly recovered from (13). Indeed, with $k = 1$, we have $\{e^{2\pi i m/k}\} = \{1\}$ and $\Delta = (e^\lambda - 1)/(\lambda e^\lambda) = g(\lambda)$ with sharing, $\Delta = 1/(1+\lambda)$ without. With $k = 2$, we have $\{e^{2\pi i m/k}\} = \{1, -1\}$ and (13) turns into (12).

Let us also consider the case of triplets, where we have $k = 3$. The set of 3th roots of unity is equal to $\{e^{2\pi i m/3}\} = \{1, (-1 + i\sqrt{3})/2, (-1 - i\sqrt{3})/2\}$, and thus, after simplification

$$\sum_{m=0}^{2} e^{\lambda e^{2\pi i m/3}} = e^\lambda + 2e^{-\lambda/2} \cos \frac{\lambda\sqrt{3}}{2}.$$

By differentiation, we find

$$\sum_{m=0}^{2} e^{2\pi i m/3} e^{\lambda e^{2\pi i m/3}} = \frac{d}{d\lambda} \sum_{m=0}^{2} e^{\lambda e^{2\pi i m/3}} = e^\lambda - e^{-\lambda/2} \left(\sqrt{3} \sin \frac{\lambda\sqrt{3}}{2} + \cos \frac{\lambda\sqrt{3}}{2} \right),$$

and

$$\sum_{m=0}^{2} e^{4\pi i m/3} e^{\lambda e^{2\pi i m/3}} = \frac{d^2}{d\lambda^2} \sum_{m=0}^{2} e^{\lambda e^{2\pi i m/3}} = e^\lambda + e^{-\lambda/2} \left(\sqrt{3} \sin \frac{\lambda\sqrt{3}}{2} - \cos \frac{\lambda\sqrt{3}}{2} \right).$$

And finally,

$$\Delta = \frac{e^\lambda + 2e^{-\lambda/2} \cos \frac{\lambda\sqrt{3}}{2} - 3}{\lambda \left(e^\lambda - e^{-\lambda/2} \left(\sqrt{3} \sin \frac{\lambda\sqrt{3}}{2} + \cos \frac{\lambda\sqrt{3}}{2} \right) \right)}, \text{ with sharing}$$

and

$$\Delta = \frac{e^{3\lambda/2} - \sqrt{3} \sin \frac{\lambda\sqrt{3}}{2} - \cos \frac{\lambda\sqrt{3}}{2}}{(\lambda + 1) \left(e^{3\lambda/2} - \cos \frac{\lambda\sqrt{3}}{2} \right) + \sqrt{3}(\lambda - 1) \sin \frac{\lambda\sqrt{3}}{2}}, \text{ without sharing.}$$

3.3 Toxicity of multiple encapsulation

Sometimes the inclusion of too many individuals (of any genotype) in a compartment can inhibit the downstream replication. This situation arises, when, for example, the compartments are dorplets of an emulsion and the genes (genomes) and their products (phenotypic manifistation) are delivered by bacterial cells. If the bacteria are lysed inside the droplets and their content is inhibiting to the subsequent steps, the replication efficiency will drop with the increase of the number of coencapsulated bacteria.

Although this case is not purely a case of P_n different from the Poisson law, and it rather modifies $\varphi(n)$, it nevertheless can be mathematically considered as modifying P_n. Indeed, in this case the regular sharing rule $\varphi(n)$ in (5) is modified by the toxicity factor $\varphi(n) \mapsto t(n)\varphi(n)$ (see (7)). However, what is imporntant is the product $P_n t(n)\varphi(n)$, therefore, one can equivalently use the modification $P_n \mapsto P_n t(n)$, keeping the sharing rule $\varphi(n)$ the same.

We will consider only two simple cases of this phenomenon: 1) when the replication is not influenced, if the number of individual does not surpass some critical number k, and it is completely inhibited otherwise, and 2) when the replication efficiency decays exponentially, $t(n) = d^{-n}$.

3.3.1 Critical toxicity

In this case we have $P_n \sim \lambda^n/n!$, when $n \leqslant k$, and $P_n = 0$ otherwise. After a simple algebraic rearrangement, using (5), we conclude

$$\Delta = \frac{e_k(\lambda) - 1}{\lambda e_{k-1}(\lambda)} \quad \text{with sharnig;} \quad \Delta = \frac{1}{1 + \lambda \frac{e_{k-2}(\lambda)}{e_{k-1}(\lambda)}} \quad \text{without sharing,}$$

where

$$e_k(x) \stackrel{\text{def}}{=} \sum_{n=0}^{k} \frac{x^n}{n!}$$

is the truncated Taylor expansion of the exponential function.

Note that $\Delta \to 1$, as $\lambda \to 0$, and $\Delta \to k^{-1}$ as $\lambda \to \infty$, as it should be. Indeed, when λ is very large, the compartments with functional replication are dominated by the ones with k individuals, and we have a convergence to the k-ploid case.

In particular, two practically interesting cases are $k = 2$ and $k = 3$ ($k = 1$ effectively means no cocompartmentalization, and $k > 3$ is difficult to justify theoretically). For $k = 2$ we have

$$\Delta = \frac{1}{2}\frac{2+\lambda}{1+\lambda} \quad \text{with sharnig;} \quad \Delta = \frac{1+\lambda}{1+2\lambda} \quad \text{without sharing,}$$

while for $k = 3$ we have

$$\Delta = \frac{1}{3}\frac{6+3\lambda+\lambda^2}{2+2\lambda+\lambda^2} \quad \text{with sharnig;} \quad \Delta = \frac{2+2\lambda+\lambda^2}{2+4\lambda+3\lambda^2} \quad \text{without sharing.}$$

3.3.2 Exponential decay

Consider that the replication efficiency decays by the same factor $d > 1$ with every individual added to a compartment. In this case we have $P_n \sim d^{-n}\lambda^n/n!$, and the resulting expressions are immediately derived noticing that this is equivalent to a simple change of λ ($\lambda \mapsto \lambda/d$)

$$\Delta = g(\lambda/d) \quad \text{with sharnig;} \quad \Delta = \frac{d}{d+\lambda} \quad \text{without sharing.}$$

4 Polydisperse droplets

Let us consider the case of random compartmentalization but with compartments that vary in size. Let us first suppose that there are in total M compartments (of all sizes) and N individuals. Then we can define the bulk occupancy

$$\tilde{\lambda} \stackrel{\text{def}}{=} \frac{N}{M}.$$

Let us in addition suppose that there are finite number of size (volume) casses of compartments. A class j is characterized by the compartment volume v_j and the number of its members M_j ($\sum_j M_j = M$). If the casses are large enough and if the individuals are homogeneously spread in the encapsulated solution, the number of individuals in all the jth class compartments is equal to $N_j = v_j M_j N / \sum_k v_k M_k$ ($\sum_j N_j = N$), that is this number constitutes the same part of the total N as the part that the volume of all the jth compartments takes in the total volume of all compartments. Let us introduce the mean number of individuals in a compartment of class j

$$\lambda_j \stackrel{\text{def}}{=} \frac{N_j}{M_j} = \frac{\tilde{\lambda}}{\bar{v}} v_j, \quad \text{where} \quad \bar{v} = \frac{\sum_j v_j M_j}{M}. \tag{14}$$

The value λ_j servs as the local Poisson parameter for the individuals redistribution law in the jth class of droplets. More specifically, the probability to find n individuals in a randomly chosen compartment of class j (with the volume v_j) is (exactly or approximately in the limit of large population and compartments number in each class, depending on the protocol of the compartmentalization) equal to $e^{-\lambda_j}\lambda_j^n/n!$.

Another observations important for the following is that if p_j is the probability for a randomly chosen *compartment* to be in class j ($p_j = M_j/M$) and q_j is the probability for a randomly chosen *individual* to be in class j ($q_j = N_j/N$), then they are related by

$$q_j = \frac{N_j}{N} = \frac{v_j}{\bar{v}}\frac{M_j}{M} = \frac{v_j}{\bar{v}}\, p_j, \tag{15}$$

or simply by $q_j \sim v_j p_j$ and, taking into account (14), $q_j \sim \lambda_j p_j$.

4.1 General expressions

Now we will generalize this to an arbitrary compratment volume distribution in the following way. Let the distribution of *compartments* by the volume be given by the probability density $\omega(v)$. For instance, the preceding example corresponds to $\omega = \sum\limits_j p_j\delta_{v_j}$. As before, we denote the mean volume of compartments by $\bar{v} = \langle\omega(v), v\rangle$. Then λ is distributed in the ensamble of *compartments* according to the probability density $F_*\omega$, where F_* is the pushforward of the function $F\colon x \mapsto x\tilde{\lambda}/\bar{v}$, so for any test function φ we have $\langle F_*\omega, \varphi\rangle = \langle\omega, \varphi\circ F\rangle$. In particular, $\tilde{\lambda}$ is the mean of $F_*\omega$. According to (15), the distribution Λ of λ in the population of *individuals* is then equal to

$$\Lambda(\lambda) = \frac{\lambda F_*\omega\,(\lambda)}{\langle\lambda F_*\omega\,(\lambda), 1\rangle} = \frac{\lambda}{\tilde{\lambda}}\, F_*\omega\,(\lambda) = \frac{\bar{v}}{\tilde{\lambda}^2}\,\lambda\omega\left(\frac{\bar{v}}{\tilde{\lambda}}\,\lambda\right). \tag{16}$$

Let us denote $\overline{\lambda^n}_\Lambda \overset{\text{def}}{=} \langle\Lambda(\lambda), \lambda^n\rangle$, and thus $\bar{\lambda}_\Lambda = \langle\Lambda(\lambda), \lambda\rangle$, and in general $\overline{\Phi(\lambda)}_\Lambda \overset{\text{def}}{=} \langle\Lambda(\lambda), \Phi(\lambda)\rangle$ (the subscript Λ is kept not to forget that all the averages are taken over the *population of individuals* and not over the ensamble of compartments). Then it is easy to show that

$$\bar{\lambda}_\Lambda = \tilde{\lambda}\frac{\overline{v^2}}{\bar{v}^2}, \quad \text{and in general} \quad \overline{\lambda^n}_\Lambda = \tilde{\lambda}^n\frac{\overline{v^{n+1}}}{\bar{v}^{n+1}}, \tag{17}$$

where $\overline{v^n}$ is the nth moment of ω: $\overline{v^n} \overset{\text{def}}{=} \langle\omega(v), v^n\rangle$.

To find Δ, we have to rewrite (7) for polydisperse compartments. The structure of the formula is the following

$$\rho'(x) = \frac{\text{mean fitness of genotype } x}{\text{mean population fitness}}\,\rho(x) = \frac{\langle w_x\rangle}{\langle w\rangle}\,\rho(x).$$

Let us denote $P_n(\lambda) \overset{\text{def}}{=} e^{-\lambda}\lambda^n/n!$. For a given subpopulation with a given λ, the mean fitness of genotype x is equal to (see [3])

$$\langle w_x(\lambda)\rangle = \frac{\sum\limits_n P_n(\lambda)n\langle\delta_x\otimes\rho^{\otimes n-1}, f_n\rangle}{\sum\limits_n P_n(\lambda)n} = \frac{1}{\lambda}\sum_n P_n(\lambda)n\langle\delta_x\otimes\rho^{\otimes n-1}, f_n\rangle,$$

while the mean subpopulation fitness is equal to

$$\langle w(\lambda)\rangle = \frac{\sum\limits_n P_n(\lambda)n\langle\rho^{\otimes n}, f_n\rangle}{\sum\limits_n P_n(\lambda)n} = \frac{1}{\lambda}\sum_n P_n(\lambda)n\langle\rho^{\otimes n}, f_n\rangle.$$

The total population mean fitness of genotype x and the total overal population fitness are obtained from these values by averaging in λ: $\langle w_x\rangle = \overline{\langle w_x(\lambda)\rangle}_\Lambda$, $\langle w\rangle = \overline{\langle w(\lambda)\rangle}_\Lambda$. Therefore, with $\rho = (1-p)\delta_0 + p\delta_x$ and with the same assumptions and using the same method, as in Section 2, in the limit $p \to 0$ we obtain

$$\Delta = \frac{\overline{\left(\lambda^{-1}\sum_n P_n(\lambda) n \varphi(n)\right)}_\Lambda}{\overline{\left(\lambda^{-1}\sum_n P_n(\lambda) n^2 \varphi(n)\right)}_\Lambda}. \tag{18}$$

We will again consider only cases $\varphi(n) = 1/n$ (sharing) and $\varphi(n) = 1$ (no sharing). With sharing, in the numerator of (18) we have $g(\lambda)$ averaged over λ, and in the denominator we have 1. Without sharing, in the numerator we have 1 and in the denomenator we have $(\lambda + \lambda^2)/\lambda = 1 + \lambda$ averaged over λ. Therefore, we have

$$\Delta = \overline{g(\lambda)}_\Lambda, \quad \text{with sharing;} \qquad \Delta = \frac{1}{1 + \bar{\lambda}_\Lambda}, \quad \text{without sharing.}$$

Let us rewrite these quantities in terms of the volume distribution in compartments ω, which is linked to Λ by (16). In the case of sharing, we have

$$\Delta = \overline{g(\lambda)}_\Lambda = \left\langle \frac{\lambda}{\tilde{\lambda}} F_* \omega\,(\lambda), \frac{1 - e^{-\lambda}}{\lambda} \right\rangle = \left\langle F_* \omega\,(\lambda), \frac{1 - e^{-\lambda}}{\tilde{\lambda}} \right\rangle = \left\langle \omega(v), \frac{1 - e^{-\tilde{\lambda} v/\bar{v}}}{\tilde{\lambda}} \right\rangle = \frac{1 - \psi_\omega(-\tilde{\lambda}/\bar{v})}{\tilde{\lambda}}, \tag{19}$$

where $\psi_\omega(y) \overset{\text{def}}{=} \langle \omega(x), e^{xy} \rangle$ is the moment generating function of ω.

The case without sharing is even simpler. Here we obtain, using (17)

$$\Delta = \frac{1}{1 + \tilde{\lambda}\, \overline{v^2}/\bar{v}^2}. \tag{20}$$

4.2 Monodispersity increases efficiency

There are two general ways of how the water-in-oil emulsion for compartmentalized evolutionary experiments is generated: 1) shaking of the bulk mixture of genome carrier containing solution and oil resulting in a significantly polydisperse emulsion and 2) generation of a monodisperse emulsion with microfluidics. As the shaking is simpler than the microfluidic approach, it is interesting to know, if there are any benefits to the latter. We will see that polydispersity (shaking) indeed worsence the selection effeciency. More precisely, of two emulsions, a polydisperse and a monodisperse ones, with the same bulk $\tilde{\lambda}$ and the same average droplet volume $\bar{v}$ (it is just the droplet volume for the monodisperse emulsion) the polydisperse emulsion always has Δ not larger than the mododisperse one.

We will only consider the case with sharing and the case without sharing, as before. In the monodisperse case we have the volume distribution given by $\delta_{\bar{v}}$. Let us denote the volume distribution of the polydisperse case by ω and let us keep all the notations associated with ω that were introduced previously. With no sharing, according to (20), we have

$$\Delta_{\text{polydisperse}} = \frac{1}{1 + \tilde{\lambda}\overline{v^2}/\bar{v}^2} \leqslant \frac{1}{1 + \tilde{\lambda}} = \Delta_{\text{monodisperse}},$$

as for any ω we have $\overline{v^2} \geqslant \bar{v}^2$.

With sharing, so called Jansen's inequality can be used, which states the following. Consider any probability distribution ρ and any convex on $\operatorname{supp}\rho$ function φ, that is for any μ such that $0 \leqslant \mu \leqslant 1$ and any x_1 and x_2 from $\operatorname{supp}\rho$ we have

$$\varphi(\mu x_1 + (1 - \mu)x_2) \leqslant \mu\varphi(x_1) + (1 - \mu)\varphi(x_2).$$

Then Jansen's theorem states that the following holds

$$\varphi(\bar{x}_\rho) \leqslant \overline{\varphi(x)}_\rho.$$

In words, the mean of the convex function is larger than the value of the function of the mean. As e^x is a convex function of x, we conclude from (19) that $\psi_\omega(y) \geqslant e^{y\bar{v}}$ and

$$\Delta_{\text{polydisperse}} = \frac{1-\psi_\omega(-\tilde\lambda/\bar v)}{\tilde\lambda} \leqslant \frac{1-e^{-\tilde\lambda}}{\tilde\lambda} = g(\tilde\lambda) = \Delta_{\text{monodisperse}}.$$

Using the fact that $g(\lambda)$ itself is a convex function, we conclude, by Jansen's inequality, that

$$\overline{g(\lambda)}_\Lambda \geqslant g(\bar\lambda_\Lambda) = g(\tilde\lambda\overline{v^2}/\bar v^2),$$

where Λ is the distribution of λ in the population for the polydisperse case (in the monodisperse case this distribution is given by $\delta_{\tilde\lambda}$). Therefore, we have an estimate from above on the gain in Δ due to switching to a monodisperse emulsion in the case of sharing

$$\Delta_{\text{mododisperse}} - \Delta_{\text{polydisperse}} \leqslant g(\tilde\lambda) - g(\tilde\lambda\overline{v^2}/\bar v^2).$$

4.3 Expressions for special distributions

Usually, the experimentally compartment size distribution is fit with some common distribution with few parameters. Therefore, it would be interesting to see, how the efficiency of selection depends on these parameters. We will assume that the compartments are spherical droplets of an emulsion. We will only consider the normal distribution, the Gamma distribution, and the lognormal distribution of both radii and volumes.

4.3.1 Normal distribution

If volumes are distributed normally with mean μ and standard deviation σ, thus with the density function

$$\omega(v) = \frac{1}{\sqrt{2\pi}\sigma}\exp\left(-\frac{(v-\mu)^2}{2\sigma^2}\right),$$

so $\bar v = \mu$, then we have

$$\Delta = \frac{1-e^{-\tilde\lambda}e^{\tilde\lambda^2\frac{\sigma^2}{2\mu^2}}}{\tilde\lambda} \quad \text{with sharing,} \qquad \Delta = \frac{1}{1+\tilde\lambda\left(1+\frac{\sigma^2}{\mu^2}\right)} \quad \text{without sharing.}$$

It must be noted that the normal distribution can adequately represent the volume distribution only for $\sigma \ll \mu$. With $\sigma \gtrsim \mu$, a significant portion of the distribution represents unphysical negative volumes.

In applications, however, it is the radii distribution which is measured instead of the volume distribution. Let the droplet radii r be distributed normally with mean radius μ_r and the standard deviation σ_r. Then, without sharing, one has

$$\Delta = \frac{1}{1+\tilde\lambda R}, \tag{21}$$

where

$$R = \frac{\overline{r^6}}{\overline{r^3}^2} = \frac{\mu_r^6 + 15\mu_r^4\sigma_r^2 + 45\mu_r^2\sigma_r^4 + 15\sigma_r^6}{(\mu_r^3 + 3\mu_r\sigma_r^2)^2}.$$

Unfortunately, the pure normal distribution of radii is not adequate for the case with sharing, as the Laplace transform of the corresponding volume distribution diverges.

4.3.2 Gamma distribution

Gamma distribution, which is given by

$$\omega(v) = \frac{v^{\alpha-1}}{\Gamma(\alpha)\theta^\alpha}e^{-v/\theta},$$

and which has two parameters, too (the shape parameter α and the scale parameter θ), has two advantages with respect to the normal distribution: it is does not have the negative part and it is skewed, which better fits empirical

cases. In this case, the mean volume is equal to $\bar{v} = \alpha\theta$, the n-th moment is equal to $\overline{v^n} = \alpha(\alpha+1)\ldots(\alpha+n-1)\theta^n$, and the Laplace transform of the distribution is equal to $\mathcal{L}[\omega](x) = \psi_\omega(-x) = (1+\theta x)^{-\alpha}$. Therefore, we obtain

$$\Delta = \frac{1 - \left(1 + \frac{\tilde{\lambda}}{\alpha}\right)^{-\alpha}}{\tilde{\lambda}} \quad \text{with sharing,} \qquad \Delta = \frac{1}{1 + \tilde{\lambda}\frac{\alpha+1}{\alpha}} \quad \text{without sharing.}$$

Note that these distributions do not depend on θ.

If the raduis, instead, is well fit with the gamma distribution

$$\varrho(r) = \frac{r^{\alpha_r - 1}}{\Gamma(\alpha_r)\theta_r^{\alpha_r}} e^{-r/\theta_r},$$

then, for the case of no sharing, Δ is given by (21) with

$$R = \frac{(\alpha_r+3)(\alpha_r+4)(\alpha_r+5)}{\alpha_r(\alpha_r+1)(\alpha_r+2)}.$$

We were not able to express Δ in a closed form for the case of sharing.

4.3.3 Lognormal distribution

Finally, many empirical distributions of the droplet size in emulsions are well fit with lognormal distribution given by

$$\omega(v) = \frac{1}{\sqrt{2\pi}v\sigma} \exp\left(-\frac{(\ln v - \mu)^2}{2\sigma^2}\right), \tag{22}$$

For this distribution, we have $\overline{v^n} = e^{n\mu + n^2\sigma^2/2}$. Therefore, we conclude that with no sharing we have

$$\Delta = \frac{1}{1 + \tilde{\lambda}e^{\sigma^2}}.$$

This value does not depend on μ.

Unfortunately, there is no known closed form expression for the Laplace transform of the lognormal distribution.

It is the radius distribution that is usually empirically fit with the lognormal distribution. However, as this distribution family is invariant under the variable chaneg of the form $x \mapsto x^\alpha$, it is only necessary to know how to derive σ for the volume distribution knowing the radial distribution. Let the raduis be distributed with the density function

$$\varrho(r) = \frac{1}{\sqrt{2\pi}r\sigma_r} \exp\left(-\frac{(\ln r - \mu_r)^2}{2\sigma_r^2}\right).$$

Then, as $v = \frac{4}{3}\pi r^3$, the volume is distributed as (22) with $\sigma = 3\sigma_r$ and $\mu = 3\mu_r + \ln\frac{4\pi}{3}$.

References

[1] Zadorin, A.S.; Rondelez, Y. Selection strategies for randomly partitioned genetic replicators. *Phys. Rev. E* **2019**, *99*, 062416.

[2] Baret, J.C.; Miller, O.L.; Taly, V.; Ryckelynck, M.; El-Harrak, A.; Frenz, L.; Rick, C.; Samuels, M.L.; Hutchison, J.B.; Agresti, J.J.; et al. Fluorescence-activated droplet sorting (FADS): efficient microfluidic cell sorting based on enzymatic activity. *Lab Chip* **2009**, *9*, 1850–1858.

[3] Zadorin, A.S.; Rondelez, Y. Natural selection in compartmentalized environment with reshuffling. *J. Math. Biol.* **2019**, *79*, 1401–1454.
